# Clarifying the taxonomy of some cryptic blennies (Blenniidae) in their native and introduced range

**DOI:** 10.1038/s41598-022-12580-z

**Published:** 2022-06-09

**Authors:** M. Pilar Cabezas, Oscar M. Lasso-Alcalá, Elena Quintero-T, Raquel Xavier, Tommaso Giarrizzo, Jorge L. S. Nunes, Fabiola S. Machado, Jesús Gómez, Wellington Silva Pedroza, Michael J. Jowers

**Affiliations:** 1 Faculdade de Ciências da Universidade do Porto, Rua do Campo Alegre s/n, 4169-007 Porto, Portugal; 2 CIBIO-InBIO, Centro de Investigação em Biodiversidade e Recursos Genéticos, Universidade do Porto, Rua Padre Armando Quintas nº 7, 4485-661 Vairão, Portugal; 3 Museo de Historia Natural La Salle, Fundación La Salle de Ciencias Naturales, Caracas, Venezuela; 4 Instituto de Ciências Do Mar (LABOMAR), Universidade Federal do Ceará (UFC), Avenida da Abolição, 3207, Fortaleza, Brazil; 5 Núcleo de Ecologia Aquática e Pesca da Amazônia (NEAP), Universidade Federal do Pará (UFPA), Belém, PA Brazil; 6 Laboratório de Organismos Aquáticos, Departamento de Oceanografia e Limnologia, Universidade Federal do Maranhão, São Luís, MA Brazil; 7 Universidad Metropolitana, Caracas, 1073 Venezuela; 8 Departamento de Biologia, Coleção de Peixes da Universidade Federal do Maranhão e Laboratório de Ecologia e Sistemática de Peixes, Universidade Federal do Maranhão, São Luís, MA Brazil; 9 Departamento de Zoología, Facultad de Ciencias, Universidad de Granada, 18071 Granada, Spain; 10 BIOPOLIS Program in Genomics, Biodiversity and Land Planning, CIBIO, Campus de Vairão, 4485-661, Vairão, Portugal

**Keywords:** Ichthyology, Speciation, Taxonomy, Molecular biology

## Abstract

*Omobranchus punctatus* is native to the Indo-Pacific region and invasive in the Atlantic region, currently being considered one of the most widely distributed blenny species. However, recent molecular studies indicated that *O. punctatus* is a complex of species, with three divergent mtDNA lineages identified to date, stressing the need for a taxonomic revision. In this study, we used an integrative approach, combining morphological and genetic data, to shed light on the taxonomy and distribution of *O. punctatus*. Moreover, we provide the first genetic records of introduced populations in Brazil and discuss the introduction pattern of this species in this region. Morphological data shows that *O. punctatus* consists of at least five distinct and geographically restricted species: *O. punctatus sensu stricto*, *O. dispar*, *O. sewalli*, *O.* cf. *kochi*, and *O.* cf. *japonicus*. Species delimitation analyses performed using the mtDNA data available confirmed that *O. punctatus *sensu stricto, *O. dispar* and *O. sewalli* correspond to different species that started to diverge about 2.6 Mya. Furthermore, *O. sewalli* was identified as the invasive species colonizing Atlantic shores. The existence of historical oceanographic barriers, such as the emergence of the Sunda Shelf in the Eastern Indian Ocean during the Pleistocene, and the biological traits of these blennies are the most likely factors responsible for their genetic differentiation and subsequent speciation.

## Introduction

Invasive alien species (IAS) constitute one of the major threats to marine biodiversity and ecosystems worldwide^1^. IAS can out-compete native species, act as facilitators of hosts and/or vectors of parasites and pathogens, change the community structure, and alter ecosystem processes, thereby impairing the associated ecosystem function. Ultimately, IAS can lead to significant economic impacts with its associated detrimental effects on human well-being^2^.

Fishes are among the most commonly introduced organisms globally^3^, with documented increases over recent decades due to globalization, changes in seawater temperatures, and the ever-increasing magnitude of shipping, aquaculture, fisheries, aquarium trade, and habitat modification (e.g., dams, canals or waterways, urbanization and deforestation)^1,4,5^. Nevertheless, most genetic and genomic studies on marine alien fishes have been mainly focused on a relatively small number of taxa^6^. In this context, blennies (Blenniidae) are among the most neglected groups of reef vertebrates^7^, despite their high invasive potential^8,9^ and being one of the most diverse families of teleost fishes (with at least 405 species widely distributed in tropical and subtropical habitats)^10,11^. As far as we know, a total of 11 blenny species have so far been introduced far from their native range (Supplementary Table S1): e.g., *Petroscirtes breviceps*^12^ to Papua New Guinea^13^, *Omobranchus anolius*^12^ to New Zealand^14^, or *Parablennius thysanius* (Jordan & Seale 1907) to the Hawaiian Islands and the Mediterranean Sea^15,16^.

**Figure 1:**
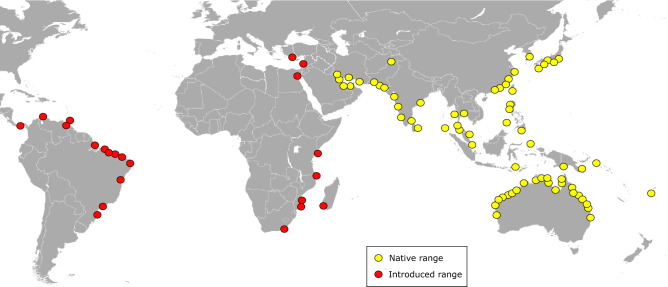
Current global distribution of *Omobranchus punctatus* group including its native (in yellow) and introduced range (in red). See Tables S2–S4 for further details for each location (year of the first record, reference collection, sources, and genetic data availability). Records of introduced populations in the Western Atlantic Ocean (WAO) would correspond to *Omobranchus sewalli*^23^, and that from the Western Indian Ocean (WIO) and the Mediterranean Sea to *Omobranchus* cf. *sewalli* (see Table 2).

*Omobranchus punctatus*^12^ is thought to be one of the most widely distributed blenny species, largely due to transport associated with ship’s ballast water and biofouling, which favour its long-distance dispersal^17–21^. It is considered native to the Indo-Pacific region, ranging from the Persian Gulf, in the Western Indian Ocean (WIO) and the Arabian Sea, to South-East Asia, Japan, Australia and the Fiji Islands, in the Western Pacific Ocean (WPO) (Fig. 1 and Supplementary Table S2, and references therein). The first occurrences of *O. punctatus* outside its native range were recorded from 1930 to 1963 in five localities on the island of Trinidad (Republic of Trinidad and Tobago) and one in Venezuela, in the Western Atlantic Ocean (WAO)^17,22,23^ (Supplementary Table S3). Since then, *O. punctatus* has been identified in another 39 localities on the Atlantic coast of Central and South America, including Panama (1966–1974, three localities), Colombia (1989, one locality), Venezuela (1978–2009, 14 localities), and Brazil (2002–2019, 21 localities) (Fig. 1 and Supplementary Table S3). Moreover, it has also been introduced to the Suez Canal, the Mediterranean and Red Sea, and along the eastern coast of Africa (Fig. 1 and Supplementary Table S4, and references therein). According to historical and morphological analyses, it seems that *O. punctatus* was first introduced to the WAO on slave boats from the Bay of Bengal (Madras or, more probably, Calcutta) to Trinidad, secondarily spreading to Venezuela, Panama and Colombia^17,21,24–26^, and then to different Brazilian localities^4,17,19,27–29^ (Supplementary Table S3).

Although there is no genetic data for this exotic blenny in the Bay of Bengal (the putative origin of invasive populations), and only one mitochondrial sequence is available from its introduced range (Venezuela)^30^ (Supplementary Table S3), recent molecular analyses showed the existence of several highly divergent genetic lineages across the species’ distribution. First, the study of Gibbs et al.^31^ showed the existence of three deeply divergent lineages within *O. punctatus*: two lineages in the Eastern Indian Ocean (EIO), separated by the Sunda Shelf Barrier (SSB), were confirmed by both mitochondrial and nuclear data; a third lineage was identified in Japan with nuclear markers, with no mtDNA data currently available for the species in this region. Later, Mehraban et al.^32^, found an additional highly divergent mtDNA lineage in the Oman Sea, which the authors suggested could correspond to the originally described *O. punctatus*, due to the proximity to the species type locality (Mumbai = Bombay, India). Recently, a phylogenetic study showed that populations outside *O. punctatus*’ native range, specifically those from the Gulf of Paria in Venezuela, clustered within the clade of individuals from the west of the SSB^30^. These data, together with the fact that the species is continuingly spreading in the Atlantic coast, highlight the urgent need to resolve *O. punctatus*’ taxonomy.

In order to continue to shed light on *O. punctatus*’ taxonomy and distribution, we conducted an integrative study of both invasive and native populations of this species, using morphological data and molecular species delimitation methods. Specifically, we aimed to: 1) confirm whether this exotic blenny represents a complex of undetected cryptic species (taxa that are morphologically similar but genetically divergent)^33,34^; and if so, 2) clarify whether they can be distinguished based on morphological characters; 3) examine their phylogenetic relationships and estimate their divergence times; and 4) unveil their distribution. In addition, we provided the first genetic records for introduced *Omobranchus* in Brazil.

## Results

### Phylogenetic data and species delimitation

The COI dataset included a total of 44 sequences, 28 from this study and 16 available from GenBank, originating from previous publications (Table 1). After quality filtering, the length of the final alignment was 560 bp. No stop codons, insertions, or deletions were observed.

**Table 1:** List of *Omobranchus* populations included in the present study. Sampling localities, source countries, geographical coordinates, number of individual sequenced (N), number of haplotypes (H), haplotype codes, references, voucher numbers and GenBank accession numbers.

Species	Locality	Country	Coordinates	N	H	Haplotype codes	Reference	Voucher Nº	COI GenBank acc. no
*Omobranchus* *punctatus* group	Araçagy, Maranhão	Brazil	2°27′54"S, 44°12′09"W	5	2	H1, H3	This study	CPUFMA 3477, 4378	OM056876-OM056880
	São Marcos, Maranhão	Brazil	2°29′28"S, 44°18′23"W	5	3	H1, H2, H3	This study	CPUFMA 3476	OM056881-OM056885
	Barra Grande, Piauí	Brazil	02°53′09.0''S, 41°38′06.1''W	6	3	H1, H2, H3	This study	GEA.ICT 04,214, 04,222	OM056858-OM056863
	Curuçá, Areuá, Pará	Brazil	00°35′05.3''S, 47°50′40.0''W	3	2	H1, H3	This study	GEA.ICT 253	OM056864-OM056866
	Jericoacoara, Ceará	Brazil	02°47′22.6''S, 40°31′11.7''W	4	1	H2	This study	GEA.ICT 12,001	OM056867-OM056870
	Salinópolis, Pará	Brazil	00°35′22.2''S, 47°19′22.3''W	5	2	H1, H3	This study	GEA.ICT 241, 242	OM056871-OM056875
	Pedernales, Orinoco Delta, Gulf of Paria	Venezuela	9°58′18.6″N, 62°15′13.7″W	1	1	H2	Cabezas et al.^30^	MHNLS 17,220	MN907119
	Andaman Sea, Trang	Thailand	7°31′16.8″ N, 99°18′18.4″E	1	1	H4	Gibbs et al.^31^	JFBM 48,521-2092	MG210393
	Qigu Harbor	Taiwan	23°8′15″N, 120°6′48.4″E	1	1	H5	Gibbs et al.^31^	JFBM 48,052-JE202	MG210394
	Gulf of Thailand, Chon Buri	Thailand	13°18′2.5″ N, 100°53′56.1″ E	2	2	H6, H7	Gibbs et al.^31^	JFBM 48,493–1999, 48,486–1969	MG210395, MG210396
	Fujian coast	China		4	4	H8, H9, H10, H11	Xu et al.^92^		KY315364, KY315361, KY315359, KY315353
	Wanwa, Miaoli	Taiwan		1	1	H12	Chang et al.^93^	ASIZP0805730	KU944802
	Xiamen, Fujian	China		1	1	H8	Xu (unpubl.)		MW518891
	Gataan, Oman Sea	Iran	25°57′31.60"N, 57°15′45.20"E	1	1	H13	Mehraban et al.^32^	ZM-CBSU 1866	MW323514
	Cabahar, Oman Sea	Iran	25°21′14.10"N, 60°36′04.50"E	1	1	H14	Mehraban et al.^32^	ZM-CBSU 1867	MW323515
*Omobranchus woodi*	Illovo stuary	South Africa	30°6′36"S, 30°51′21.6"E	3	2	H15, H16	Steinke et al.^94^	ADC08 Smith 235.30#1, ADC_235.30#2, ADC_235.30#3	JF494019, HQ561537, HQ561538

**Figure 2:**
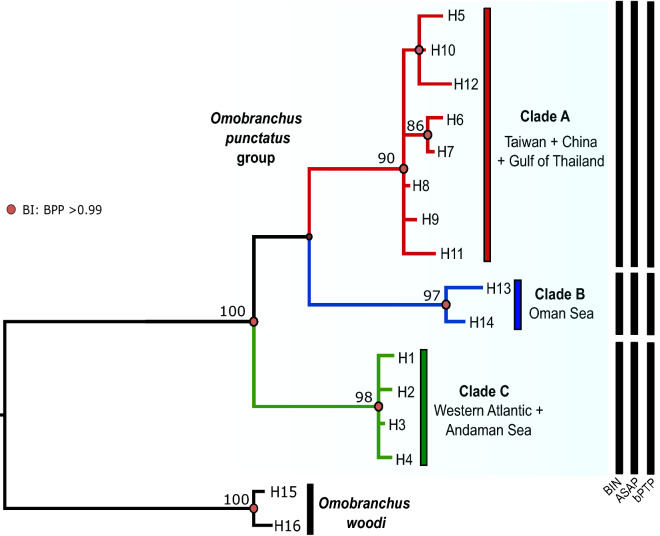
Bayesian consensus tree of *Omobranchus punctatus* group, based on COI sequences. Bayesian posterior probabilities (BPP) over 0.99 are represented by red circles at nodes and values correspond to bootstrap support (>75%) given by the maximum likelihood analyses. Clades A-C are identified. The tree was rooted with *O. woodi* (sequences available in GenBank: JF494019, HQ561537, HQ561538). Vertical black bars represent results from the species delimitation analyses: Barcode Index Number (BIN), Assemble Species by Automatic Partitioning (ASAP) and Bayesian Poisson Tree Process model (bPTP).

Phylogenetic analyses based on BI and ML approaches, rendered trees with similar overall topologies, with main clades receiving high bootstrap and posterior probabilities (> 90% and 0.99 respectively, Fig. 2). Both analyses supported the monophyly of *O. punctatus* group and the existence of three highly divergent lineages: the first one including Taiwan, China, and the Gulf of Thailand populations (Clade A), the second one restricted to the Oman Sea (Clade B), and the third one including Western Atlantic (Venezuela and Brazil) and Andaman Sea (Thailand) populations (Clade C). Mean K2P distance between these clades ranged from 4.9 to 6.0%, with the lowest value found between Clades A and C, and the highest between Clades B and C. Genetic divergence between *O. punctatus* clades and the congeneric species *O. woodi* was on average 11.8% (ranging from 10.8 to 12.4%).

**Figure 3:**
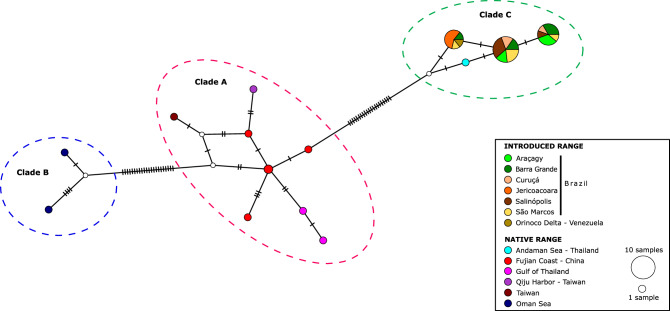
Median-joining network of all COI sequences for the *Omobranchus punctatus* group. Localities are coded by filling patterns (see legend). Each circle represents a haplotype, and its size is proportional to the observed haplotype frequency. Non-observed haplotypes are represented by small white circles. Every crossbeam on the connecting lines between haplotypes represents a single mutational step. Distinct clades (A-C) are depicted as dashed-lines circles.

The minimum spanning network also supported the existence of three divergent groups within *O. punctatus* (Fig. 3), which matched the distinct clades observed in the phylogenetic analyses (Fig. 2). A total of 14 haplotypes were identified: 8 in Clade A, two in Clade B and four in Clade C. From these haplotypes, 10 were unique and only four (H1-H3 and H8) were shared by more than one individual. Within each clade, most haplotypes were separated by few mutational steps. However, clades were separated by at least 21 mutational steps (Fig. 3). Overall, 11 haplotypes (H4-H14), most of them restricted to a single location, were identified in the native range at the Indo-Pacific region (WIO, EIO, WPO); meaning that only 3 haplotypes were found among introduced populations of the Atlantic coast of Central and South America (Fig. 3, Table 1). No haplotypes were shared between native and introduced populations. Nevertheless, the haplotype from the Andaman Sea population (H4) was separated by only one nucleotide substitution from the most abundant haplotype found in Brazil (H3). On the other hand, within the three haplotypes observed in the introduced populations, only the haplotype H2 was shared between Brazilian and Venezuelan individuals.

Finally, all species delimitation analyses (BIN, ASAP and bPTP) clustered the sequences of *O. punctatus* in 3 distinct MOTUS (Fig. 2). These results are in agreement with those from phylogenetic analysis and therefore, strongly support the existence of three distinct species.

### Evolutionary divergence

**Figure 4:**
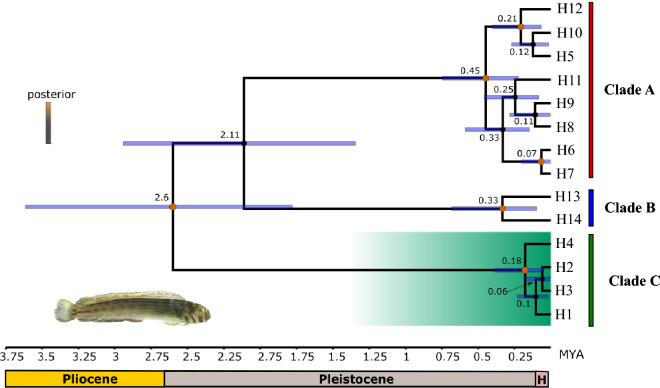
Bayesian time tree for *Omobranchus punctatus* group as inferred by BEAST. Scale bar in Mya. The green clade represents the samples sequenced in this study. Clades A-C are identified. Bayesian posterior probabilities are represented by colour and node size (red values by nodes are strongly supported). Values by nodes indicate the estimated age of the split event and horizontal blue bars represent 95% of the highest posterior density (HPD) interval. H denotes Holocene. Photo of *O. punctatus* from Venezuela by James Van Tassell (American Museum of Natural History) and Ross Robertson (Smithsonian Tropical Research Institute).

The MCC tree (Fig. 4) recovered a sister clade relationship of Western Atlantic haplotypes (and Andaman Sea) (Clade C) to all other haplotypes (Clades A and B) and was highly similar to the BI topology (Fig. 2). Posterior probabilities higher than 98% were recovered for all but one main node. Based on a mutation rate of 1.2% per Mya, which is commonly used to date divergence between fish^35–37^, the estimated divergence times between Clade C and rest of haplotypes was 2.6 Mya (1.7–3.6, 95% HPD). The estimated divergence time between the Clade B, including Oman Sea populations, and the rest of the native haplotypes (Clade A) was of 2.1 Mya (1.3–2.9, 95% HPD). Divergence time between populations from the Western Atlantic (haplotypes H1, H2 and H3) and the Andaman Sea (haplotype H4) was estimated at 180,000 years ago (0.05–0.39, 95% HPD), within the late Pleistocene, whereas divergences times within western Atlantic haplotypes occurred within the early Holocene. Time to most recent common ancestor for haplotypes belonging to Clades A and B was estimated at 0.45 Mya (0.22–0.75, 95% HPD) and 0.33 Mya (0.1–0.68, 95% HPD), respectively.

### Morphological analysis and species nomination

**Figure 5:**
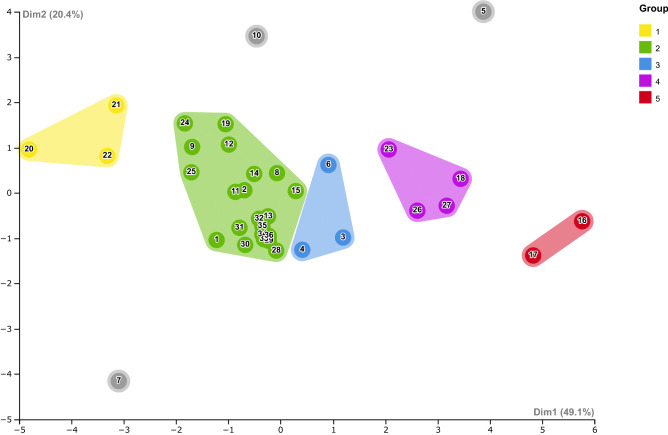
Principal Component Analysis (PCA) based on 10 meristic characters analysed in 36 populations (localities) of *Omobranchus punctatus* group. 1: *Omobranchus* cf. *kochi*, 2: *Omobranchus sewalli*; 3: *Omobranchus punctatus sensu stricto*, 4: *Omobranchus* cf. *japonicus*, and 5: *Omobranchus dispar*. Populations analysed are listed in Table S6. First (Dim 1) and second (Dim 2) principal components accounted for nearly 70% of the total variance.

In the PCA based on 10 meristic characters of 584 individuals identified as *O. punctatus*, the first two principal components (PC1 – 49.09%; PC2 – 20.44%) accounted for nearly 70% of the total variance (Supplementary Table S5). This analysis clustered the individuals into 5 morphogroups (Fig. 5), 3 of them matching the genetic clades observed in the phylogenetic analyses. Morphogroup 1 included all individuals from Papua New Guinea, and Salomon and Moluccas islands, all of them in the WPO (Fig. 5, Supplementary Table S6). Morphogroup 2, the biggest one and which corresponds to the genetic Clade C (Fig. 2), grouped individuals from 21 locations belonging to the 4 regions analysed in the present study (WIO, EIO, WPO and WAO) (Fig. 5, Supplementary Table S6). Interestingly, all Western Atlantic introduced populations (Panama, Trinidad, Venezuela, and Brazil) were included in this group, which also included native populations from the Andaman Sea (Nicobar Islands and west coast of Thailand) and the Gulf of Bengal (Sri Lanka and Vizagapatam) in the EIO (Fig. 5, Supplementary Table S6). Morphogroup 3 consisted of individuals from the WIO, specifically from the Gulf of Oman, the Arabian Sea, and the type locality of Bombay (Fig. 5, Supplementary Table S6). This group corresponds to the Clade B in the phylogenetic analyses (Fig. 2). Morphogroup 4 included individuals from two Australian populations together with those from Japan and the Fiji Islands (Fig. 5, Supplementary Table S6). This morphogroup could correspond to the clade that included an individual from Japan recovered by Gibbs et al.^31^ based on nuclear genetic data. Finally, the last morphogroup, which corresponds to the genetic Clade A (Fig. 2), included Chinese populations (Hong Kong and Zhoushan Island) located at the Fujian Coast (Fig. 5, Supplementary Table S6).

Both genetic and morphological results supported the existence of at least three distinct species within the *O. punctatus* group. Thus, following the Priority Principle of the ICZN and considering the existent synonyms and their corresponding type locality (Supplementary Table S7), the different clades/morphogroups were identified as follow (Table 2). The genetic Clade A or morphogroup 5 corresponds to *Omobranchus dispar*^38^, whose type locality is Amoy (now Xiamen) at the Fujian coast (Supplementary Table S7). The Clade B or morphogroup 3 should be considered the true *Omobranchus punctatus*, hereafter *O. punctatus*^12^ *sensu stricto* (Table 2), since it included the Bombay population, the type locality of this nominal species (Supplementary Table S7). Finally, Clade C or morphogroup 2 corresponds to *Omobranchus sewalli*^23^ (Table 2), a species first described in Trinidad (Supplementary Table S7). Although further molecular analyses should be conducted to confirm the species status of the remaining morphogroups (1 and 4), they could correspond to *Omobranchus* cf. *kochi*^39^ (type locality = mouth of the Meranke River at Papua New Guinea) (Supplementary Table S7) and *Omombranchus* cf. *japonicus*^40^ (type locality = Tokyo, Japan) (Supplementary Table S7), respectively.

**Table 2:** Genetic clade, morphological group, and type localities corresponding to each of the three proposed species (*Omobranchus punctatus sensu stricto*, *Omobranchus dispar* and *Omobranchus sewalli*) within *Omobranchus punctatus* group.

Proposed species	Genetic Clade – Morphogroup	Type localities		
		Springer and Gomon (1975)	Current	Present study
*Omobranchus punctatus sensu stricto*^12^	B – 3	Mumbai (West coast, India)	Bombay (West coast, India)	Bombay (West coast, India)
		Gulf of Oman	Gulf of Oman	Oman Sea
*Omobranchus dispar*^38^	A – 5	Amoy (China)	Xiamen (China)	Fujian Coast (China)
		Zhoushan Island	Zhoushan (North of Taiwan)	China
				Qigu Harbor (Taiwan)
		Gulf Thailand (North Coast)	Gulf Thailand (North Coast)	Gulf Thailand (North Coast)
*Omobranchus sewalli*^23^	C – 2	Thailand (West Coast)	Thailand (West Coast)	Andaman Sea
		Nikobar Island	Andaman and Nikobar Island	Andaman and Nikobar Island
		Trinidad (West Indies)	Trinidad	Trinidad

## Discussion

In this study we used an integrative approach, using morphological and the most comprehensive genetic dataset available to date (mtDNA), to shed light on the taxonomy and worldwide distribution of the *Omobranchus punctatus* group. Our morphological analysis suggests the existence of five species within this group, three of which could be genetically confirmed by the present study, and another one supported by the genetic data of Gibbs et al.^31^. Although further molecular and morphological analysis are required to fully understand the distribution of these species, genetic and morphological data were generally congruent. The present results also suggest that only *Omobranchus sewalli* is found beyond the Indo-West Pacific region, the native range of the genus.

### Species delimitation

Although *Omobranchus* Valenciennes^12^ is the most species-rich genus in the Omobranchini tribe of the Blenniidae, with 23 valid species^24,41^, its actual diversity remains underestimated^7,30–32^.

Our phylogenetic analyses are in agreement with those of Gibbs et al.^31^, Cabezas et al.^30^ and Mehraban et al.^32^, recovering three well differentiated and supported mitochondrial lineages within *O. punctatus* group (Clades A, B and C) (Figs. 2 and 3), which were also confirmed by three different approaches of species delimitations methods (Fig. 2). The genetic divergences recovered were slightly lower as compared to what has been established in earlier studies on blennies species^32,42^. However, they were greater or equal tenfold to the mean intraspecific distance established in the seminal work by Hebert et al.^43^ and exceed the threshold value of 3% established for species delimitation in fishes^44^. On the other hand, morphological analyses based on meristic characters discriminated five distinct morphogroups (Fig. 5), three of them corresponding to the genetic lineages recovered by the phylogenetic analyses. Unfortunately, for the remaining two groups no mitochondrial data were available. As in previous studies^24^, no single character could be discerned to distinguish between morphogroups, something that seems to be common in small, cryptobenthic fishes where most species can only be distinguished through combinations of different morphological traits^7,42^. To further understand which characters could be used in species diagnosis, a further extensive and in-deep morphological analysis, with more specimens, would be needed. Overall, the concordance between molecular and morphological groups, strongly supports that the muzzled blenny *Omobranchus punctatus* is a species complex consisting of at least three distinct species, which are geographically restricted.

The use of integrative taxonomic approaches (combining morphological, genetic, and ecological data, among other characters) has shown to be the best strategy to produce well-supported species delimitations^45,46^. However, most studies concentrate exclusively on documenting current species diversity or identifying independent lineages without naming them^46^, as is also the case for previous studies on *O. punctatus*^30–32^. The proper naming of the detected lineages is essential in biodiversity assessments and for its subsequent conservation planning^45^, especially when dealing with IAS^47^. Therefore, following the ICZN and considering the list of synonymies attributed to *O. punctatus*^24,41^ (Supplementary Table S7), we were able to identify each phylogenetic/morphological group (Table 2). According to our results, specimens from the first genetic Clade (Clade A) and the morphogroup 5, mainly distributed in China (Figs. 2, 3 and 5), can be attributed to *Omobranchus dispar*^38^ (Table 2), a species originally described as *Petroscirtes dispar* Günther, 1861 from the Chinese locality of Amoy (Xiamen) (Supplementary Table S7). As observed in previous molecular studies^30,32^, Clade A also included specimens sequenced from Gulf of Thailand and Taiwan localities (Figs. 2 and 3). The analyses of meristic characters, however, placed them in the morphogroup 2 (Fig. 5; Supplementary Table S6), probably due to morphological stasis^48^. For this reason, although Taiwan and Thailand specimens could belong to *O.* cf. *dispar* (see Supplementary Table S7), such taxonomical arrangement will require an additional morphological and genetic analysis.

The second clade (Clade B) and its corresponding morphogroup 3 were restricted to the WIO (Figs. 2, 3 and 5). Since these included the specimens from Bombay (type locality) and nearby localities from the Oman Sea, we designated it as *Omobranchus punctatus sensu stricto* (hereafter *O. punctatus s.s.*) (Table 2), in accordance with what was concluded in the study by Mehraban et al.^32^. Although genetic data for Bombay is lacking, this locality could be the source population for *O. punctatus* in the Oman Sea, due to the ocean currents pattern in this region, and which could explain the morphological affinities found between these populations (Fig. 5). The complex oceanographical conditions in the region^49,50^ would allow the connectivity between *O. punctatus* populations along the WIO (North Arabian Sea), ensuring a significant level of gene flow and, therefore, preventing speciation. In fact, other records of this blenny species have been documented from the Arabian Sea (Fig. 1, Supplementary Table S2), such as in the Gulf of Kutch (India) and the coast of Karachi (Pakistan), where it was described as *Salarias sindensis* (Day, 1888), currently considered a synonym of *O. punctatus s.s.* (Supplementary Table S7).

The Clade C, and its corresponding morphogroup 2, showed a wider distribution (Figs. 2, 3 and 5; Supplementary Table S6). All populations from the WAO, including those from Brazil, sequenced and morphologically analysed for the first time in the present study, were included in this third lineage. The Andaman Sea (Thailand) population from the EIO (Bay of Bengal) was also included in this clade (Figs. 2 and 3), thus agreeing with recent phylogenetic studies^30,32^. Morphogroup 2 included a larger number of populations from all the four oceanic regions (WIO, EIO, WPO and WAO) (Fig. 5; Supplementary Table S6). The population of Trinidad, the first record of “*O. punctatus*” in WAO^24^, was also included in this group, therefore supporting the morphological affinity previously found between WAO and EIO populations^24^. Considering all the above, we assigned Clade C and morphogroup 2 to the species *Omobranchus sewalli*^23^ (Table 2), originally described as *Poroalticus sewalli* by Fowler^23^ from tide pools of the west coast of Trinidad in the WAO (Supplementary Table S7). Interestingly, *O. sewalli* is the only species of the genus that has been described based on an introduced population^23^. To the best of our knowledge, this could be one of few cases where an introduced fish was described as a new species^23^, later synonymized (Supplementary Table S7)^24,41^, and posteriorly confirmed as a species through to morphological and genetic data (present study).

Finally, morphological analyses retrieved two additional groups (morphogroups 1 and 4), for which no mitochondrial data are available (Fig. 5; Supplementary Table S6). Morphogroup 1 included populations from Papua New Guinea and nearby islands, and could correspond to *Omobranchus kochi*^39^ (referred herein as *Omobranchus* cf. *kochi*, Supplementary Table S7), a species first described from the Meranke River in southern New Guinea (Supplementary Table S7). On the other hand, morphogroup 4 included Japan, Australia, and Fiji Islands populations (Fig. 5; Supplementary Table S6). Because this group may correspond in part, to the highly divergent genetic lineage found by Gibbs et al.^31^ based on nuclear data from individuals collected in Kagoshima, Japan, we assigned these specimens to *Omobranchus* cf. *japonicus*^40^ (Supplementary Table S7). However, further molecular analyses are necessary to confirm the taxonomic status of fishes from these two regions.

### Phylogeographic and ecological insights

The fact that most benthic marine species have planktotrophic (self-feeding) larvae that can spend days to months in the water column, has led marine ecologists to presume that most marine populations are demographically “open” and therefore naturally highly connected^51,52^. However, many studies in the last decade have confirmed this perception to be inaccurate^42,53^, with oceanographic processes (e.g., oceanographic barriers, currents, habitat discontinuities) and the biological traits of species (e.g., dispersal abilities, larval duration) being the main likely mechanisms responsible of population differentiation and their subsequent speciation^52,53^.

In the present study, the geographic distribution of the three lineages reported for the *O. punctatus* group, now recognized as three distinct species (*O. dispar*, *O. punctatus s.s.*, and *O. sewalli*), indicates that their limited dispersal abilities but also the existence of oceanographic barriers played an important role in the differentiation of these species. In the EIO and WPO, the separation between *O. sewalli* and *O. dispar*, based on a mutation rate of 1.2% per Mya^35–37^, seems to have occurred at the beginning of the Pleistocene (~ 2.6 Mya; Fig. 4), coinciding with the emergence of the SSB, a phylogenetic break located at the Thai-Malay Peninsula (TMP)^54^. The emergence of the SSB due to sea-level lowering during Plio-Pleistocene glaciations restricted gene flow between the tropical Indian Ocean and the WPO, which possibly led to isolation among populations and their further differentiation^55^. Therefore, allopatric speciation seems to be the most likely scenario to explain the separation between *O. sewalli* and *O. dispar* in this region. In fact, the presence of this phylogeographic break separating populations from the Andaman Sea from those located West of the TMP has been suggested in previous studies of these species^30–32^. Moreover, it has also been documented for other fishes^56^, including other *Omobranchus* species^31^, as well as for many other marine taxa, including sharks^57^, crustaceans^58^, and molluscs^59^. Interestingly, specimens from the Gulf of Thailand were morphologically more closely related to those on the west side of the TMP than to WPO individuals (Fig. 5). Long-lasting extreme environmental conditions, as those from estuaries, intertidal areas, and tide pools that *Omobranchus* species inhabit, may have prevented morphological differentiation by imposed stabilizing selection on morphology^48^. Further molecular and morphological analyses including additional populations from both regions are necessary to resolve the inconsistency found between both type of analyses, which could indicate the possible coexistence of both lineages and/or the existence of hybrids.

On the other hand, as suggested by Mehraban et al.^32^, different hydrological and ecological characteristics prevalent in the Arabian Sea and the Bay of Bengal (Andaman Sea) could explain the differentiation between *O. punctatus s.s* and *O. sewalli* ( Figs. 2, 3, 4 and 5, Table 2 and Supplementary Table S6), inhabiting the Indian Ocean, and that occurred at approximately 2.6 Mya (Fig. 4). Populations inhabiting divergent environments deal with different selection pressures during their evolution, which determine their geographic distribution, and enhance ecological speciation^60^.

In addition, the life history traits of *Omobranchus* species could also have favoured the differentiation of *O. punctatus s.s*, *O. dispar*, and *O. sewalli*. The species of this genus are small, benthic inhabitants of intertidal zones and tide pools in coastal marine and estuarine ecosystems^17,20,24,61,62^. They are considered as permanent residents, showing a strong site fidelity during most of their life^20,21,27,61,63,64^. Moreover, their fertilized eggs are adhesive and demersal, and larvae are planktonic, settling about 3–7 weeks after hatching^65^ usually in protected areas near to the coast (OML-A and JLSN personal observations). All these factors suggest that *Omobranchus* species have limited dispersal capabilities (by natural means)^64,66^, which could have affected the connectivity among populations, and, thus, promoted their genetic differentiation and subsequent speciation.

### Introduction pattern of Omobranchus sewalli

Of the three species proposed in the present study, only *O. sewalli* occurs outside the Indo-West Pacific region (Fig. 5; Supplementary Tables S3, S6, and S7), the natural distribution range considered for *Omobranchus* species^24,41^. Indeed, *O. sewalli* is the only species of the genus recorded in the WAO (Supplementary Table S7)^24,41^. Considering the limited natural dispersal capabilities of this species, as discussed above, the presence of *O. sewalli* in this very distant geographical area could be explained as a result of human-mediated activities, which, intentionally or unintentionally, transport species beyond their natural ranges^67^ (see Supplementary Tables S1 and S3).

Supported by historical, morphological, and recent molecular analyses^17,24,26,30–32^, the Andaman Sea has been suggested as the most likely source population of *O. sewalli* in the WAO. The present results (Figs. 2, 3 and 5) also confirm this. In Brazil, the first occurrences of *O. sewalli* were registered in the states of Rio de Janeiro, Bahia, and Santa Catarina (Supplementary Table S3). Since then, the species spread rapidly to many other northern and southern localities, mainly by ballast water, biofouling, oil rigs and larval dispersal on nearshore ocean currents (see Supplementary Table S3and references herein). Nevertheless, due to the lack of molecular data, the source population and introduction pattern of *O. sewalli* in this region remained unknown until now. In the present study, we provided the first genetic records of this species for Brazil. Based on mitochondrial data, three haplotypes (H1–H3) were observed in this region (Table 1, Fig. 3). The presence of haplotype H2 in São Marcos, Barra Grande and Jericoacoara, which is also present in Venezuela (Table 1, Fig. 3), indicate that: 1) *O. sewalli* could have been introduced to Brazil directly from a Venezuelan population; or 2) the same pathway may have been responsible for the introduction of *O. sewalli* in Venezuela and Brazil. In addition, the exclusive presence of haplotypes H1 and H3 in all Brazilian populations, except for Jericoacoara (Table 1, Fig. 3), suggest that more than one introduction pathway may been operating in this region. Unfortunately, due to the limited genetic data available for the WAO no robust conclusions can be reached.

Human-mediated dispersal also seems to be responsible for the introduction of this blenny in the east coast of Africa (Mozambique, Madagascar, Tanzania, Kenya, and South Africa (Fig. 1, Supplementary Table S4) ^9,17^. The main arguments are: 1) the limited natural dispersal capabilities of this species; 2) the ocean currents pattern existing in the region; and 3) the remoteness from the closest native populations (likely the Andaman Sea – 7000 km). The morphological analyses conducted in the present study grouped Mozambique, Andaman Sea and WAO populations together (morphogroup 2, Fig. 5; Supplementary Table S6), suggesting a close relationship between them and indicating that they could be the same species. For this reason, we suggest that Mozambique (previously described as *Omobranchus japonicus scalatus* by Smith 1959) and the remaining African populations of this blenny should be considered as *Omobranchus* cf. *sewalli* (Supplementary Table S7), pending further confirmation.

## Conclusions

This is the first study to perform an integrative analysis combining morphological and genetic data on the *Omobranchus punctatus* group. Our data suggests the existence of five species within this group, with *O. sewalli* identified as the invasive species colonising Atlantic shores. Considering the association of *O. sewalli* with man-made vectors, its high tolerance to a wide range of salinity levels and adverse environmental conditions^9,17,18,21^, and its capability of self-recruitment^19,64^, further introductions of this species are likely expected. Recent records of *O. sewalli* in Brazil (see Supplementary Table S3), confirm this assumption.

## Methods

### Sample collection

**Figure 6:**
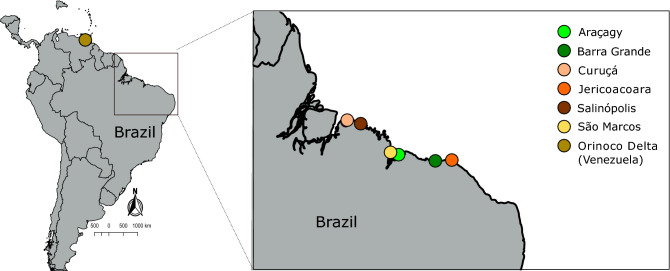
Sampling locations of the putative species *Omobranchus punctatus* (species considered in this work as *Omobranchus sewalli*) along the Atlantic coast of South America. See Table 1 for additional information.

Between 2012 and 2021, a total of 28 specimens of the putative species *O. punctatus*, were collected, using hand nets and anaesthetic clove oil^63^, from intertidal flat reefs at six localities along the Brazilian coast (Fig. 6, Table 1 and Supplementary Table S3). For each specimen, a small fragment of muscle and fin tissue was removed and stored in 96% ethanol for the molecular analyses. Voucher specimens were fixed in 10% formalin, and later stored in 70% ethanol and deposited at the Fish Collection of the Universidade Federal de Maranhão (CPUFMA-UFMA), under the numbers CPUFMA 3476–3478, and at the Universidade Federal do Pará (Aquatic Ecology Group – GEA.ICT) under the numbers GEA.ICT#241, 242, 253, 04214, 04222 and 12001 (Table 1). Samples were collected with the permission of the Instituto Chico Mendes de Conservacão da Biodiversidade (ICMBio) and the Sistema de Autorizacão e Informacão em Biodiversidade (SISBIO), license numbers 67481 and 35625, respectively. The care and use of experimental animals complied with Brazilian animal welfare laws, guidelines, and policies. No surgical procedures were performed, and no procedures that cause lasting harm to the fish were carried out. All methods conducted were in accordance with ARIIVE guidelines.

### DNA extraction, PCR amplification, and sequencing

Total genomic DNA was extracted from a small amount of alcohol-preserved muscle tissue using the Purelink Genomic DNA Mini Kit (Invitrogen, Paisley, UK), according to the manufacturer’s protocol. A fragment (~ 670 bp) of the mitochondrial cytochrome c oxidase subunit I (COI, standard barcoding fragment) was amplified by polymerase chain reaction (PCR) using the M13 tailed primer cocktail C_FishF1t1 and C_FishR1t1^68^. PCR amplifications consisted of 25 µl reaction volumes containing 3–5 µl of template DNA, 10 × buffer MgCl_2_ free (Invitrogen), 2 mM MgCl_2_, 0.2 mM dNTPs, 1 µM of each primer, 0.3 U Platinum Taq DNA polymerase (Invitrogen), and double-distilled H_2_O to volume. PCR conditions used were as described in Cabezas et al.^30^.

The resulting PCR products were purified and bidirectionally sequenced at GENEWIZ (Leipzig, Germany).

### Sequence analysis and phylogenetic reconstructions

All newly obtained sequences were edited with Sequencher v5.4.6 (Gene Codes Corporation, Ann Arbor, MI, USA), and checked for potential contaminations using GenBank’s BLASTn search^69^. They were thereafter deposited in GenBank (see Table 1).

All COI sequences of *O. punctatus* (N = 13) available from GenBank (November 2021) were included in the phylogenetic analysis. Additionally, COI sequences of the closely related species *O. woodi* (Gilchrist & Thompson, 1908) were used as outgroups (see Table 1). Sequences were aligned using MUSCLE^70^ as implemented in MEGA X^71^. The final dataset was checked for the presence of pseudogenes by translating sequences into amino acids.

Phylogenetic tree reconstructions were performed using maximum likelihood (ML) and Bayesian inference (BI), through Garli v2.0.1^72^ and MrBayes v3.2.6^73^, respectively. Only one individual (or sequence) per haplotype was included in the phylogenetic analyses to reduce redundancy. Analyses were conducted using data partitions by codons (1 + 2 + 3) to minimize saturation effects of codon positions and to account for different rates of evolution of each one. The Akaike Information Criterion (AICc)^74^ implemented in PartitionFinder v2.1.1^75^ was used to select the best fit evolutionary model for each partition. The resulting models were SYM (1st position), F81 + I (2nd position) and GTR + G (3rd position). ML analysis was performed using 10 independent searches and 1,000 bootstrap replicates. The evaluation of log-likelihood values across searches allowed to check the convergence between the topologies of the trees generated. The SumTrees command from the package DendroPy^76^ was used to summarize non-parametric bootstrap support values for the best tree, after generating a majority-rule consensus tree. For the BI analysis, two independent runs (each with four Markov chains for 2 × 10^7^ generations) were performed. Trees and parameters were sampled every 1,000 generations, with the heating parameter set to 0.25. The convergence of the analyses was validated by the standard deviation of split frequencies being lower than 0.01 and by graphical monitoring of the likelihood values over time using Tracer v1.7.1^77^. The majority-rule consensus tree was estimated combining results from duplicated analyses, after discarding 25% of the total samples as burn-in. Clades with bootstrap support or BI posterior probability (BPP) greater than 90% or 0.9, respectively, were considered well supported. The consensus tree inferred for each phylogenetic approach was visualized and rooted using FigTree v1.4.4^78^, and later prepared as a graphic with the software Inkscape v1.0.1 (http://www.inkscape.org). Pairwise nucleotide distances among clades were calculated using the Kimura-2-Parameter model (K2P)^79^ implemented in MEGA X. In addition, relationships among haplotypes were further examined by building a median-joining network using the PopART v1.7 software^80^.

### Molecular species delimitation

Analyses of species delimitation were performed on the COI dataset using three different approaches: two distance-based methods, the Barcode Index Number (BIN) system^81^ and the Assemble Species by Automatic Partitioning (ASAP)^82^; and one tree-based method, the Bayesian Poisson Tree Process (bPTP) model^83^. Using the BIN system of the Barcode of Life Data Systems (BOLD)^84^, COI sequences were clustered into molecular operational taxonomic units (MOTUs) independent of any prior taxonomic assignment, and then assigned to a unique alphanumeric code or BIN. This method provides a means of confirming the concordance between barcode sequence clusters and species designations^81^. The ASAP method was implemented on a web interface (https://bioinfo.mnhn.fr/abi/public/asap/asapweb.html), and it was applied with default settings using the K2P distance matrix. By building partitions from single locus sequence alignments, it provides a score for each defined partition and sorts the sequences into putative species^82^. Finally, the bPTP model was performed on the PTP species delimitation web server (https://species.h-its.org/ptp/), using the Bayesian tree as input, running 100,000 MCMC generations, and with the burn-in set to 25%. In contrast to BIN and ASAP, bPTP infers putative species based on a non-ultrametric phylogenetic tree, mainly by identifying the transition points between inter- and intraspecific branching events^83^.

### Estimation of divergence times

Divergence times of the *Omobranchus* lineages were computed in BEAST v2.6.3^85^ together with the bModelTest package^86^. For this analysis, the dataset was reduced to unique haplotypes and a constant coalescent model was used. We used a 1.2% divergence per million years, as previously estimated for the fish COI locus^35^, and which is commonly used in marine fish studies^36,37^. Two BEAST MCMC chains were run independently with 40 M generations each, sampling every 4,000 states, and discarding the first 10% of samples as burn-in. Convergence and parameter mixing were verified with Tracer v1.7.1^77^, ensuring consistency across runs and that most parameters had sufficient effective sample sizes (ESS > 200). Trees and logfiles of both runs were then combined using LogCombiner v2.6.3, and TreeAnnotator v2.6.3 was used to summarize estimates into a maximum-clade-credibility (MCC) tree. The blenny *O. woodi* was used as an outgroup in all analyses. Trees were visualized and edited in FigTree v.1.4.4^78^. All analyses were performed on CIPRES^87^.

### Morphological analysis

For morphological analysis, previously published morphological data on 444 specimens from 29 populations of the putative species *O. punctatus*, from both its natural and introduced distribution range (see Table 13 in^24^), were combined with data collected for the present study on 140 specimens from Venezuela (N = 75) and Brazil (N = 65) (Supplementary Table S6). The specimens examined in the present study included fish that were genetically analysed (see Table 1), and others deposited in the following collections: Museo de Historia Natural La Salle (MHNLS), Museo Oceanológico Hermano Benigno Román (MOBR-EDIMAR), and Museo de Ciencias Naturales de la UNELLEZ (MCNG) in Venezuela; and CPUFMA-UFMA and GEA.ICT in Brazil. For each specimen, and following the procedure established by Springer and Gomon^24^, the 10 most important meristic characters (counts of body structures) of the genus were assessed: number of dorsal-fin spines, number of segmented rays in the dorsal and anal fins, sum of the unsegmented and segmented rays (i.e., the total dorsal-fin elements), sum of dorsal and ventral procurrent rays in the caudal fins, number of precaudal and caudal vertebrae, total number of vertebrae, number of lateral-line tubes, and the position of the last lateral-line tube relative to a dorsal-fin spine (Supplementary Table S6).

To determine the relationships among individuals from the different populations, a principal component analysis (PCA) was performed in R^88^, using the FactoMineR^89^ and factoextra^90^ packages. Prior to analysis, the frequencies (62 variations) of the 10 meristic characters analysed (discrete data) were transformed to weighted averages.

### Species nomination

The genetic lineages and associated morphological groups found in the present work (see “Results” Section) were named according to the 12 currently available synonyms of *O. punctatus*^24,41^ (Supplementary Table S7), following the Priority Principle established in article 23, Chapter 6, of the International Code of Zoological Nomenclature^91^. For this, geographical proximity between the type localities of the species described and the currently synonyms available within the group attributed to *O. punctatus* (Supplementary Table S7) is required.

## Supplementary Information

Supplementary Information 1.

Supplementary Information 2.

Supplementary Information 3.

Supplementary Information 4.

Supplementary Information 5.

Supplementary Information 6.

Supplementary Information 7.

Supplementary Information 8.

## Data Availability

The dataset generated during the current study are available in the GenBank database (Accession Numbers: OM056858—OM056885).
