# Supplementary material for: Clarifying the taxonomy of some cryptic blennies (Blenniidae) in their native and introduced range
Source: Sci Rep. 2022 Jun 9;12:9514. doi: 10.1038/s41598-022-12580-z (PMC9184548; doi:10.1038/s41598-022-12580-z)
Supplement: Supplementary file 1 — Supplementary Information 1. [file 41598_2022_12580_MOESM1_ESM.pdf]

Supplementary Information

Table S1. Records of introductions of Blenniidae species, including native distribution, date of record, locations, likely introduction vectors, and sources. Information based on references herein and the Web sources: FishBase, GBIF, VerNet, and FIshNet2. (BW, ballast water; SF, ship fouling; L, larval dispersal; OR, oil rigs; TC, transoceanic channels; GC, global change)

Table S2. Records of *Omobranchus punctatus* group in its native range, including (if known) date of reporting, reference collection, sources, and GenBank accession numbers (if available). Information based on references herein and the Web sources: GBIF, VerNet, and FIshNet2. (WIO, Western Indian Ocean; EIO, Eastern Indian Ocean; WPO, Western Pacific Ocean)

Table S3. Records of introductions of *Omobranchus punctatus* group in the Western Atlantic Ocean (WAO), including possible mechanisms of introduction, collection lots, sources and GenBank accession numbers (if available). Species considered in this work as *Omobranchus sewalli* (Fowler 1931). Information based on references herein and the Web sources: GBIF, VerNet, and FIshNet2. (BW, ballast water; SF, ship fouling; L, larval dispersal; OR, oil rigs)

Table S4. Records of introductions of *Omobranchus punctatus* group in the Western Indian Ocean (WIO) and Mediterranean Sea (MS), including possible mechanisms of introduction, collection lots, and sources. Species considered in this work as *Omobrachus* cf. *sewalli* (Fowler 1931). Information based on references herein and the Web sources: GBIF, VerNet, and FIshNet2. (BW, ballast water; SF, ship fouling; L, larval dispersal; TC, transoceanic channels; GC, global change)

Table S5. Eigenvalues, variance (%) and cumulative variance (%) of the Principal Component Analysis (Fig. 5), based on 10 meristic characters (Average Weighted), from 36 localities of *Omobranchus punctatus* group.

Table S6. Frequency distributions of the 10 meristic characters analysed (counts), and their weighted averages (AW), in 36 localities of *Omobranchus punctatus* group along its entire distribution range. Data from Venezuela and Brazil localities were obtained in the present study, and that from the remaining 29 localities were obtained from Springer & Gomon (1975).

Table S7. List of synonyms, type localities and current status proposed for the distinct species of the *Omobranchus punctatus* group. Information based on Springer & Gomon (1975), Williams (2014) and Fricke *et al*. (2021).
