# Supplementary material for: Clarifying the taxonomy of some cryptic blennies (Blenniidae) in their native and introduced range
Source: Sci Rep. 2022 Jun 9;12:9514. doi: 10.1038/s41598-022-12580-z (PMC9184548; doi:10.1038/s41598-022-12580-z)
Supplement: Supplementary file 6 — Supplementary Information 6. [file 41598_2022_12580_MOESM6_ESM.pdf]

Table S5. Eigenvalues, variance (%) and cumulative variance (%) of the Principal Component Analysis (Fig. 5), based on 10 meristic characters (Average Weighted), from 36 localities of *Omobranchus punctatus* group.

Component	Eigenvalues	% of Variance	Cumulative % of Variance
Dim.1	4.909	49.089	49.089
Dim.2	2.044	20.435	69.525
Dim.3	1.149	11.488	81.013
Dim.4	0.901	9.011	90.025
Dim.5	0.581	5.807	95.832
Dim.6	0.184	1.843	97.674
Dim.7	0.126	1.259	98.933
Dim.8	0.078	0.78	99.713
Dim.9	0.025	0.251	99.964
Dim.10	0.004	0.036	100.000
