# Supplementary material for: Clarifying the taxonomy of some cryptic blennies (Blenniidae) in their native and introduced range
Source: Sci Rep. 2022 Jun 9;12:9514. doi: 10.1038/s41598-022-12580-z (PMC9184548; doi:10.1038/s41598-022-12580-z)
Supplement: Supplementary file 8 — Supplementary Information 8. [file 41598_2022_12580_MOESM8_ESM.pdf]

Table S7. List of synonyms, type localities and current status proposed for the distinct species of the *Omobranchus punctatus* group. Information based on Springer & Gomon (1975), Williams (2014), and Fricke *et al*. (2022), and the original descriptions listed below.

**Nominal species**	**Type locality**	**Current status proposed**
Blennechis punctatus Valenciennes, 1836	Mumbai (Bombay), India	*Omobranchus punctatus* (Valenciennes 1836)*
Petroscirtes dispar Günther, 1861	Amoy (Xiamen), China	*Omobranchus dispar* (Günther, 1861)
Petroscirtes semilineatus Kner, 1868	Kandavu Island, Fiji Islands	*Omobranchus* cf. *japonicus* (Bleeker, 1869)
Petroskirtes japonicus Bleeker, 1869	Tokyo, Japan	*Omobranchus* cf. *japonicus* (Bleeker, 1869)
Salarias decipiens De Vis, 1884	Cardwell Island, Queensland, Australia	*Omobranchus* cf. *japonicus* (Bleeker, 1869)
Salarias helenae De Vis, 1884	St. Helena Island, Moreton Bay, Queensland, Australia	*Omobranchus* cf. *japonicus* (Bleeker, 1869)
Salarias sindensis Day, 1888	Karachi, Pakistan	*Omobranchus punctatus* (Valenciennes 1836)
Aspidontus dasson Jordan & Snyder, 1902	Wakanoura, Wakayama Prefecture, Japan, Inland Sea	*Omobranchus* cf. *japonicus* (Bleeker, 1869)
Petroscirtes kochi Weber, 1907	Meranke River, southern New Guinea	*Omobranchus* cf. *kochi* (Weber, 1907)
Poroalticus sewalli Fowler, 1931	Tide pools at Brighton Beach, Trinidad, West Indies	*Omobranchus sewalli* (Fowler 1931)
Petroscirtes masyae Smith, 1934	Tidepool on Koh Chula, Gulf of Thailand, off southeastern Thailand	*Omobranchus* cf. *dispar* (Günther, 1861)
Omobranchus japonicus scalatus Smith, 1959	Delagoa Bay, southeastern Mozambique, western Indian Ocean	*Omobranchus* cf. *sewalli* (Fowler 1931)

**Omobranchus punctatus* (Valenciennes, 1836) (in Cuvier and Valenciennes 1836)

Original descriptions

Cuvier, G. & Valenciennes A. Histoire naturelle des poissons. Tome onzième. Livre treizième. De la famille des Mugiloïdes. Livre quatorzième. *De la famille des Gobioïdes*. 11 (i-xx) 1-506pp. Pls. 307-343 (1836).

Günther, A. 1861. Catalogue of the fishes in the British Museum. Catalogue of the acanthopterygian fishes in the collection of the British Museum. Gobiidae, Discoboli, Pediculati, Blenniidae, Labyrinthici, Mugilidae, Notacanthi. *London*. **3**, 1-586 (1861).

Kner, R. Über neue Fische aus dem Museum der Herren Johann Cäsar Godeffroy & Sohn in Hamburg. (IV. Folge). *Sitzungsberichte der Kaiserlichen Akademie der Wissenschaften. Mathematisch-Naturwissenschaftliche Classe*. **58 (1-2)**, 26-31 (1868).

Bleeker, P. Neuvième notice sur la faune ichthyologique du Japon. Verslagen en Mededeelingen der Koninklijke Akademie van Wetenschappen. *Afdeeling Natuurkunde*. **3**, 237-252 (1869).

De Vis, C. W. New fishes in the Queensland Museum. No. 4. *Proceedings of the Linnean Society of New South Wales*. **9**, 685-698 (1884).

Day, F. Observations on the fishes of India. Part I. *Proceedings of the Zoological Society of London*. **3**, 258-265 (1888).

Jordan, D. S. & Snyder J. O. A review of the blennoid fishes of Japan. *Proceedings of the United States National Museum*. **25**, 441-504 (1902).

Weber, M. Süsswasserfische von Neu-Guinea. Ein Beitrag zur Frage nach dem früheren Zusammenhang von Neu-Guinea und Australien. in *Nova Guinea. Résultats de l'expédition scientifique Néerlandaise à la Nouvelle-Guinée en 1903* (ed. Wichmann, A.). 201-267 (1907).

Fowler, H. W. Fishes obtained by the Barber Asphalt Company in Trinidad and Venezuela in 1930. *Proceedings of the Academy of Natural Sciences of Philadelphia*. **83**, 391-410 (1931).

Smith, H. M. Contributions to the ichthyology of Siam. IX-XIX. *Journal of the Siam Society, Natural History*. **Supplement 9**, 287-325 (1934).

Smith, J. L. B. Fishes of the families Blenniidae and Salariidae of the western Indian Ocean. *Ichthyological Bulletin, Department of Ichthyology, Rhodes University*. **14**, 229-252, Pls. 14-19 (1959).
